# Orbitofrontal cortex control of striatum leads economic decision-making

**DOI:** 10.1038/s41593-023-01409-1

**Published:** 2023-08-17

**Authors:** Felicity Gore, Melissa Hernandez, Charu Ramakrishnan, Ailey K. Crow, Robert C. Malenka, Karl Deisseroth

**Affiliations:** 1 Department of Bioengineering, Stanford University, Stanford, CA USA; 2 Department of Psychiatry and Behavioral Sciences, Stanford University, Stanford, CA USA; 3 Nancy Pritzker Laboratory, Stanford University, Stanford, CA USA; 4 Howard Hughes Medical Institute, Stanford University, Stanford, CA USA

**Keywords:** Reward, Decision

## Abstract

Animals must continually evaluate stimuli in their environment to decide which opportunities to pursue, and in many cases these decisions can be understood in fundamentally economic terms. Although several brain regions have been individually implicated in these processes, the brain-wide mechanisms relating these regions in decision-making are unclear. Using an economic decision-making task adapted for rats, we find that neural activity in both of two connected brain regions, the ventrolateral orbitofrontal cortex (OFC) and the dorsomedial striatum (DMS), was required for economic decision-making. Relevant neural activity in both brain regions was strikingly similar, dominated by the spatial features of the decision-making process. However, the neural encoding of choice direction in OFC preceded that of DMS, and this temporal relationship was strongly correlated with choice accuracy. Furthermore, activity specifically in the OFC projection to the DMS was required for appropriate economic decision-making. These results demonstrate that choice information in the OFC is relayed to the DMS to lead accurate economic decision-making.

## Abstract (web summary)

The authors show that choice information is relayed from the ventrolateral orbitofrontal cortex to the dorsomedial striatum to lead accurate economic decision-making.

## Main

Economic decision-making, the process of evaluating options in the environment to inform the best course of action, is critical for a wide range of behaviors essential for survival and well-being. To make optimal decisions, the neural representation of each option must be integrated with information about the type and scale of outcome it predicts to provide a representation of the subjective value of each alternative. Representations of subjective value can then be compared before engaging neural circuits that generate flexible behavioral responses^1–3^.

Neural representations of subjective value have been identified in the orbitofrontal cortex (OFC)^4^, and electrical microstimulation of the OFC can bias choice behavior^5^. These results have supported a widespread hypothesis that the OFC has a role in economic decision-making^1,6–11^. However, lesions and inactivation of the OFC yielded conflicting results on choice behavior^12–16^. Furthermore, representations of subjective value exist in other brain regions including the medial prefrontal cortex^17^, dorsomedial striatum (DMS)^18^ and mediodorsal thalamus^19^; similar manipulations of each of these brain regions influence decision-making behavior^20–24^. Thus, multiple brain regions may have important roles in economic decision-making; however, surprisingly little is known about if and how these brain regions may interact to mediate economic choices. One reason for this limited understanding is that most studies examining the neural correlates of value-based decision-making have been conducted in nonhuman primate systems, wherein tools are more restricted for recording and manipulating activity of precisely defined populations of neurons. To address this limitation, we adapted an economic decision-making task for rats, which permits recording and manipulation of neural activity in multiple defined neural populations while rats make economic decisions.

## Results

### Integration of reward quantity and quality information during economic decision-making

**Fig. 1:**
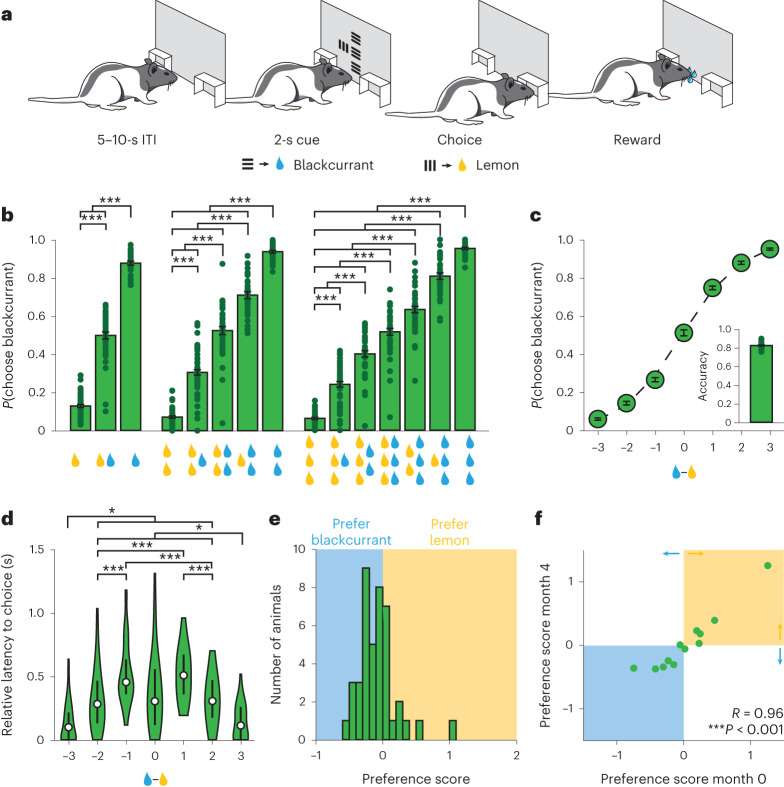
Rats integrate information about reward quantity and reward identity to make economic decisions. **a**, Schematic of economic decision-making task for rats. **b**, Probability of choosing the blackcurrant-predictive cue for all cue combinations (*n* = 42 rats, one-way repeated-measures analysis of variance (ANOVA)). **c**, Probability of choosing the blackcurrant-predictive cue as a function of difference in size of the reward available. Rats were more likely to choose the larger available reward (*n* = 42 rats, one-way repeated-measures ANOVA). Inset, fraction of trials in which the animal chose the larger available reward (*n* = 42 rats, 0.82 ± 0.01). **d**, Latency to choice nosepoke response as a function of the difference in the size of the reward available. Rats were faster when the difference in reward volume was high (easy trials) (*n* = 42 rats, one-way repeated-measures ANOVA). The center dot represents the median, the bars represent the first and third quartiles. **e**, Histogram of preference scores: the difference in available reward at which the animal was equally likely to choose the blackcurrant-predictive or lemon-predictive cue (negative values, blue shading: rats preferring blackcurrant; positive values, yellow shading: rats preferring lemon; *n* = 42 rats). **f**, Correlation of preference scores computed on sessions performed 4 months apart. Preference scores were highly correlated, indicating that the juice preferences of individual animals were stable across time (*n* = 12 rats, Pearson correlation). *P < 0.05, ***P < 0.001. Unless otherwise noted, data are presented as the mean ± s.e.m. Full statistical details are shown in Supplementary Table 1.

**Extended Data Fig. 1:**
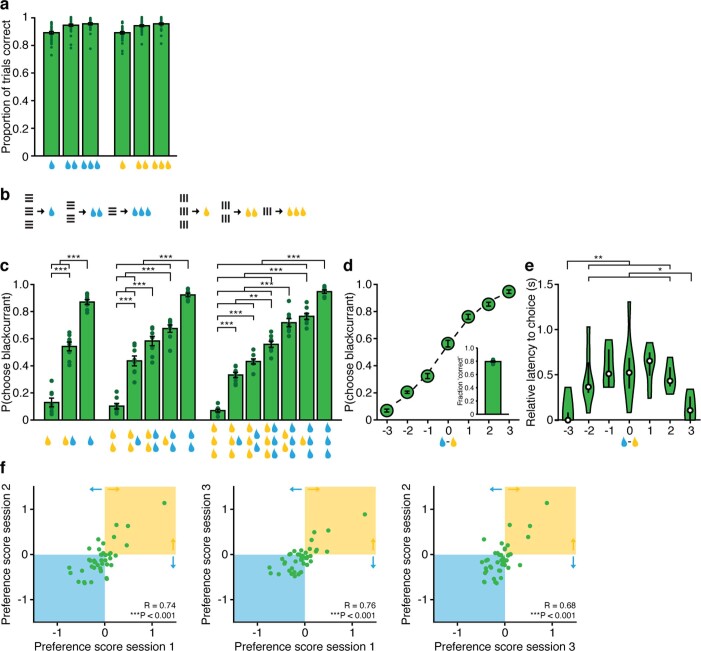
Behavioral characterization. **a**. Proportion of correct responses to each cue in final 3 training sessions before surgery; cues were comparably learned, n = 36 rats. **b**. Reinforcement contingencies where size of stimulus was not proportional to reward size for probing whether animals perform value-based or perceptual decision-making. **c**. Probability of choosing blackcurrant-predictive cue for all cue combinations for animals training using reversed reinforcement contingencies (n = 8 rats, one-way repeated-measures ANOVA). **d**. Probability of choosing blackcurrant-predictive cue as a function of the difference in the size of rewards available for animals trained using reversed reinforcement contingencies (n = 8 rats, one-way repeated-measures ANOVA). *Inset*, fraction of trials in which animal chose the larger available reward (n = 8 rats, 0.79±0.01). **e**. Latency to choice nosepoke response as a function of difference in the size of rewards available for animals trained using reversed reinforcement contingencies (n = 8 rats, one-way repeated-measures ANOVA). Center dot represents median, bars represent first and third quartile. **f**. Correlations of preference scores computed on 3 consecutive sessions. Preference scores are highly correlated, indicating juice preferences of individual animals are stable across time (Pearson’s correlation). * P < 0.05, ** P < 0.01, *** P < 0.001, Unless otherwise noted, data are presented as mean ± SEM.

In the initial experiments, we developed and validated an economic decision-making task in rats (Fig. 1a). On each trial, rats were presented with two visual cues side by side. The type of stimulus (vertical or horizontal drifting gratings) indicated the identity of the associated reward (blackcurrant-flavored or lemon-flavored water), and the size of the visual stimulus indicated the size of the associated reward. After 2 s, the animals could perform a nosepoke to the side of the chosen cue to indicate choice, whereupon the chosen reward was delivered. We found that rats reliably chose visual stimuli that predicted larger volume rewards (Fig. 1b,c). In addition, animals displayed slower choice latencies on trials in which the difference in available reward volume was small (difficult trials), compared with trials in which the difference in available reward volume was large (easy trials) (Fig. 1d). To confirm that animals were making decisions based on the value of the stimuli presented, as opposed to simply detecting larger visual cues more reliably, we included a subset of animals in which the size of the visual stimulus was not positively correlated with the size of the reward it predicted. These animals still reliably chose stimuli that predicted larger volume rewards, indicating that animals used information about the available reward volume to make appropriate decisions (Extended Data Fig. 1b–e).

We next asked whether animals used information about reward identity, in addition to information about reward volume, to guide their decision-making. For each animal, we generated a preference score by calculating the difference in available reward (the number of drops of blackcurrant-flavored water − number of drops of lemon-flavored water) at which the animal was equally likely to choose the blackcurrant-predictive or lemon-predictive cue. We found that individual animals displayed modest preferences for either blackcurrant-predictive or lemon-predictive cues (Fig. 1e). Moreover, we found that the preference scores of individual animals were strongly correlated across consecutive sessions (Extended Data Fig. 1f) as well as across sessions separated by approximately 4 months (Fig. 1f). Thus, juice preferences were stable across both short and long timescales in individual animals. Animals therefore integrated individual (subjective) internal preferences regarding the available reward quality with externally accessible (objective) information about available reward quantity to make economic decisions.

### Activity in both OFC and DMS is necessary for economic decision-making

**Fig. 2:**
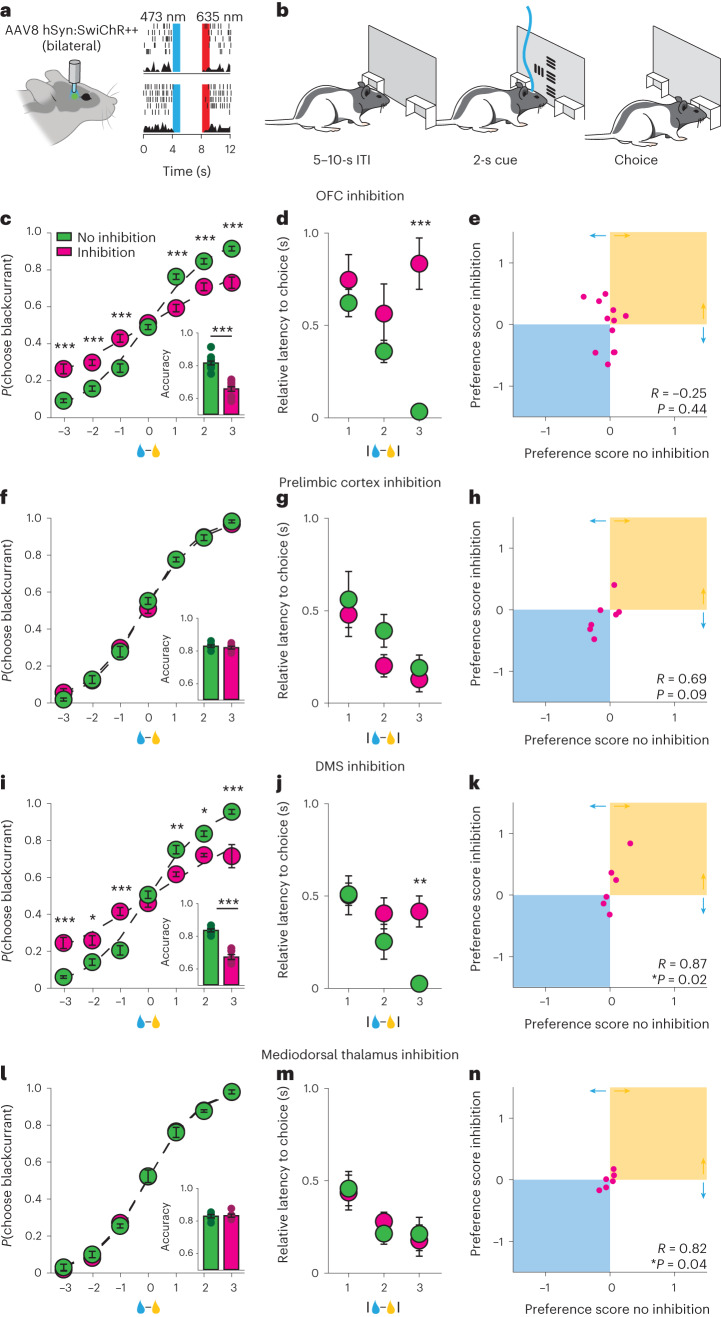
Activity in the OFC and DMS is important for economic decision-making. **a**, Left: schematic of surgical preparation. Right: example single units showing inhibition of spiking activity in response to optical stimulation. **b**, Schematic of choice task with optical inhibition restricted to the cue evaluation period. **c**–**n**, OFC and DMS inhibition impairs economic decision-making. **c**,**f**,**i**,**l**, Probability of choosing the blackcurrant-predictive cue as a function of the difference in the volume of available rewards for uninhibited (green) and inhibited (magenta) trials. Rats were less likely to choose larger volume rewards when the OFC (**c**) or DMS (**i**) was inhibited but not when the prelimbic cortex (**f**) or mediodorsal thalamus (**l**) was inhibited (OFC: *n* = 12 rats; prelimbic cortex: *n* = 7 rats; DMS: *n* = 6 rats; mediodorsal thalamus: *n* = 6 rats; two-way repeated-measures ANOVA). Inset, fraction of trials in which the animal chose the larger available reward on uninhibited (green) and inhibited (magenta) trials (two-sided paired *t*-test). **d**,**g**,**j**,**m**, Latency to nosepoke choice response as a function of the absolute difference in the size of rewards available on uninhibited (green) and inhibited (magenta) trials. Rats were slower to respond when the OFC (**d**) or DMS (**j**) was inhibited in trials wherein the difference in reward volume was high (easy trials) (OFC: *n* = 12 rats; prelimbic cortex: *n* = 7 rats; DMS: *n* = 6 rats; mediodorsal thalamus: *n* = 6 rats, two-way repeated-measures ANOVA). Inhibition of either the prelimbic cortex (**g**) or mediodorsal thalamus (**m**) did not alter response latency. **e**,**h**,**k**,**n**, Juice preferences computed on trials in which the OFC (**e**) was inhibited were not correlated with juice preferences computed on trials in which the OFC was not inhibited (Pearson correlation). Inhibition of the prelimbic cortex (**h**), DMS (**k**) or mediodorsal thalamus (**n**) did not change juice preferences. *P < 0.05, **P < 0.01, ***P < 0.001. Data are presented as the mean ± s.e.m. Full statistical details can be found in Supplementary Table 1.

**Extended Data Fig. 2:**
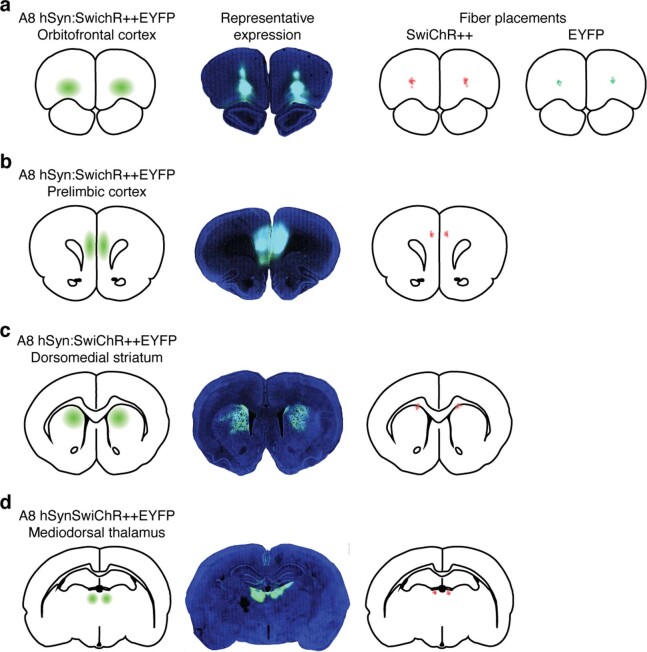
Fiber placements. **a-d**. Representative images of EYFP expression and fiber placements in animals injected with AAV8 hSyn:SwiChR++EYFP or AAV8 hSyn:EYFP in the OFC (**a**), the prelimbic cortex (**b**), the DMS (**c**), or the mediodorsal thalamus (**d**).

Electrophysiological recordings identified several brain regions that appear to encode important features of economic decision-making tasks^4,17–19^. To determine which brain regions were critical for this task, we performed an optogenetic inactivation screen. Specifically, after rats achieved criterion performance (Methods), we injected an adeno-associated virus (AAV) encoding the inhibitory stabilized step function opsin SwiChR++^25^ under the control of the human synapsin promoter (AAV8 hSyn:SwiChR++EYFP) bilaterally into the OFC, DMS, mediodorsal thalamus or prelimbic cortex, and positioned optical fibers above each of these structures (Fig. 2a and Extended Data Fig. 2). When animals had reestablished criterion performance, we asked whether optical inhibition of each of these brain structures altered decision-making performance (Fig. 2b).

**Extended Data Fig. 3:**
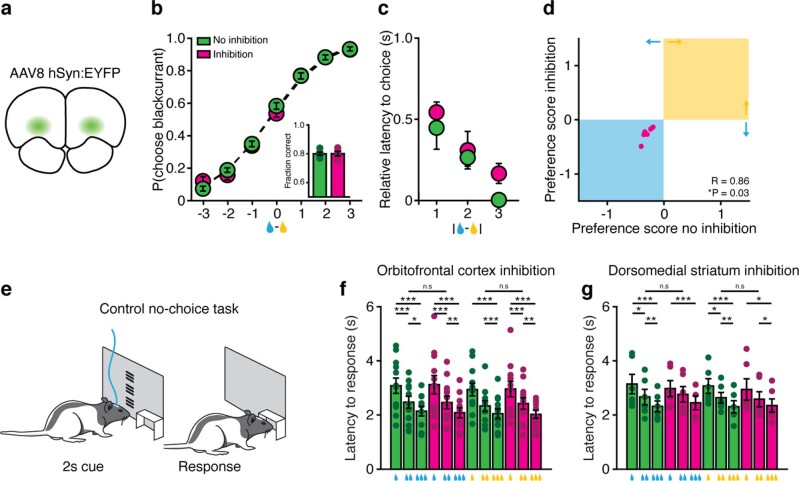
Optogenetic inhibition control experiments. **a-d**. Optical stimulation does not alter economic decision-making in animals expressing EYFP in the OFC. **a**. Schematic of experimental preparation. **b**. Probability of choosing blackcurrant-predictive cue as a function of difference in the volume of available rewards for no illumination (green) and illumination (magenta) trials (n = 6 rats, two-way repeated-measures). *Inset*, fraction of trials in which animal chose the larger available reward on no illumination (green) and illumination (magenta) trials (n = 6 rats, two-sided paired t-test). **c**. Latency to choice nosepoke response as a function of the absolute difference in the size of rewards available on no illumination (green) and illumination (magenta) trials (n = 6 rats, two-way repeated-measures ANOVA). **d**. Juice preferences computed on trials with OFC illumination are correlated with juice preferences computed on trials without OFC illumination (Pearson’s correlation). **e**. Schematic of control task for probing whether effects of optical inhibition specifically impact choice behavior. **f, g**. Latency to nosepoke response for cues predicting different size rewards in control no-choice task on trials in which the OFC (**f**) or DMS (**g**) was not inhibited (green) or was inhibited (magenta). OFC or DMS inhibition did not alter latencies to respond in no-choice control task (OFC: n = 12 rats, DMS< n = 6 rats, two-way repeated-measures ANOVA). * P < 0.05, ** P < 0.01, *** P < 0.001, Data are presented as mean ± SEM.

In accordance with previous work in mice^16^, optogenetic inhibition of the OFC impaired economic decision-making. We found that psychometric curves were flatter and latencies for easy choices were slower (Fig. 2c,d). In addition, we found that preferences computed on trials in which the OFC was inhibited were not correlated with preferences computed on trials in which the OFC was not inhibited, indicating that optogenetic inhibition of the OFC also disrupted juice preferences (Fig. 2e). Optogenetic inhibition of the DMS also impaired economic decision-making; psychometric curves were flatter and latencies for easy choices were slower, but choice preferences were unchanged (Fig. 2i–k), suggesting that decision-making based on reward volume was disrupted but juice preferences remained intact. In contrast, optogenetic inhibition of either the prelimbic cortex (Fig. 2f–h) or mediodorsal thalamus (Fig. 2l–n) had no discernible effect on economic decision-making. Decision-making was also unchanged in animals injected with control virus encoding enhanced yellow fluorescent protein (EYFP) and subjected to the same procedures (Extended Data Fig. 3a–d).

To determine whether the effects we observed were attributable to a specific deficit in economic decision-making or due to an unanticipated nonspecific effect of intervention (such as impaired visual perception, action execution or value recall), we placed the same animals into a control task, in which the choice component of the economic decision-making task was removed (Extended Data Fig. 3e). On uninhibited trials, animals were faster to respond to cues that predicted larger volume rewards, suggesting that animals could perceive the cues, remember their values and act accordingly. Importantly this relationship was maintained when either the OFC (Extended Data Fig. 3f) or DMS (Extended Data Fig. 3g) was inhibited. Thus, inhibition of the OFC or DMS impairs economic decision-making without impairing visual perception, action execution or the representation (or recollection) of cue value.

### Choice-related activity in the OFC precedes choice-related activity in the DMS

**Fig. 3:**
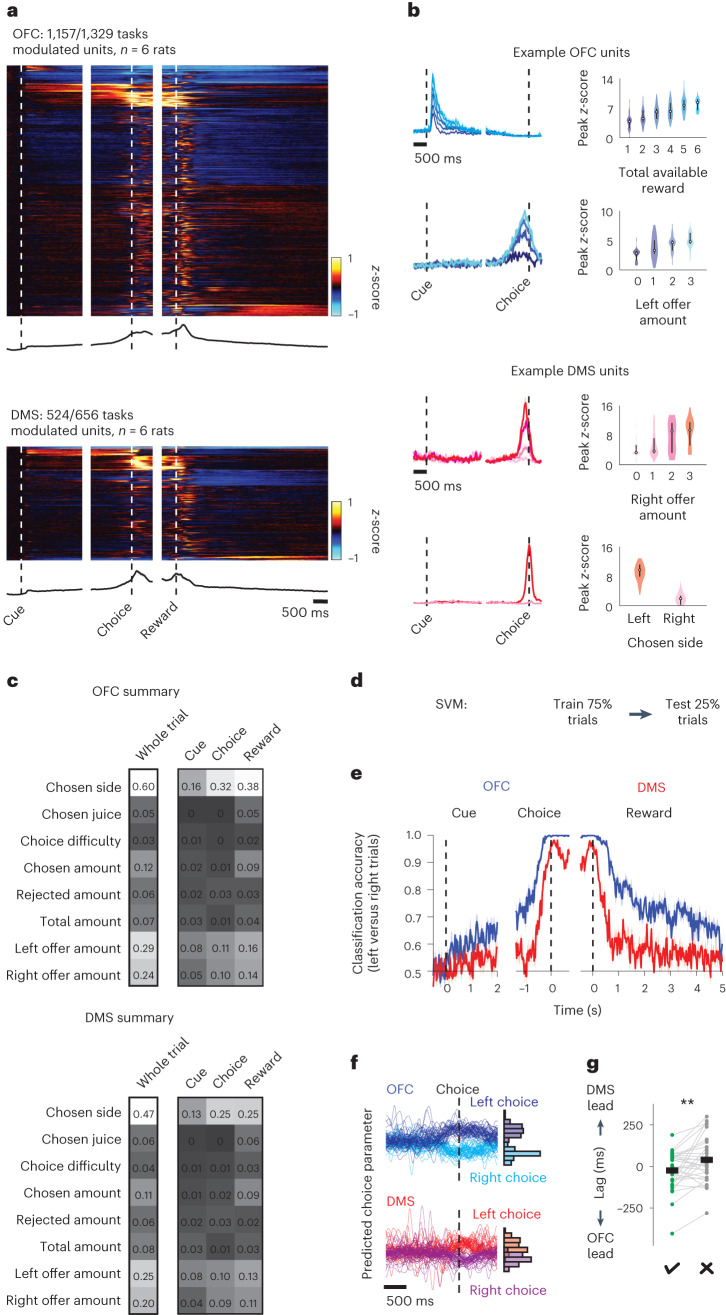
Activity in the OFC and DMS encodes spatial features of economic decision-making. **a**, Upper: heatmap of *z*-scored firing rates, averaged across trials, for each task-modulated unit in the OFC (top) or DMS (bottom). Lower: population-averaged *z*-scored firing rates. **b**, Tuning of example single units recorded in the OFC (top) or DMS (bottom). Left: trial-averaged peristimulus time histograms. Right: violin plots of peak *z*-score. Different shades of blue and red correspond to different trial types. The center dot represents the median; the bars represent the first and third quartiles. **c**, Proportion of units significantly modulated by different task features in the OFC (top) or DMS (bottom). OFC and DMS activity properties are similarly dominated by spatial features. **d**, Linear decoding approach. **e**, Chosen side classification accuracy of fourfold cross-validated SVM trained on single-unit activity in the OFC (blue) or DMS (red). Increase in decoding accuracy in the OFC precedes increase in decoding accuracy in the DMS (*n* = 656 units per brain area from six rats). **f**, Left: predicted choice parameter computed on single trials from an example single session in which activity in the OFC (top) and DMS (bottom) was recorded simultaneously. The predicted choice parameter was defined as the perpendicular distance of the decoded decision value from the support vector. Predicted choice parameters were aligned to choice response and color-coded according to the side chosen by the animal on each trial. Right: histograms of predicted choice parameters at the time when choice was indicated (*n* = 132 trials, *n* = 11 OFC units, *n* = 11 DMS units) **g**, Single-session mean peak cross-correlation lags of the OFC and DMS predicted choice parameters on trials in which the animal chose the larger available reward (correct trials, green) and trials in which the animal chose the smaller available reward (incorrect trials, gray) (*n* = 30 sessions from five rats, two-sided paired *t*-test; the black lines denote the means). **P < 0.01. Unless otherwise noted, data are presented as the mean ± s.e.m. Full statistical details are shown in Supplementary Table 1.

**Extended Data Fig. 4:**
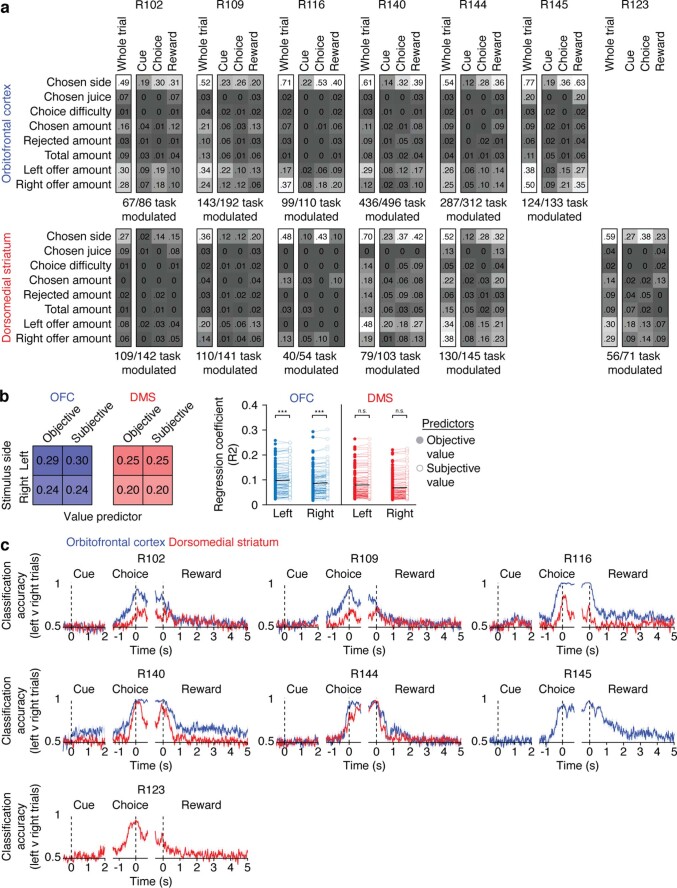
Electrophysiology supporting data 1. **a**. Proportion of units significantly modulated by distinct task features in the OFC (top) or DMS (bottom) for each individual animal. **b**. Left, proportions of neurons significantly modulated by objective (reward size) and subjective value did not differ across OFC (blue) or DMS (red). Right, coefficients of determination (R2) of each modulated unit in either OFC (blue) or DMS (red) when either the subjective or objective value of the stimuli presented on either left or right were used as predictors. Black lines denote means. OFC units were more strongly modulated by subjective value than objective value (n = 107 units per condition, three-way mixed ANOVA). **c**. Chosen-side classification accuracy of 4-fold cross validated support vector machine trained on single unit activity in the OFC (blue) or DMS (red) for each individual animal (R102 n = 86 units per brain area, R109 n = 141 units per brain area, R116 n = 54 units per brain area, R140 n = 103 units per brain area, R144, n = 145 units per brain area, R145 n = 133 OFC units, R123 n = 71 DMS units). * P < 0.05, ** P < 0.01, *** P < 0.001, Data are presented as mean ± SEM.

To explore in more detail what function these brain areas might have in economic decision-making, we performed wireless extracellular electrophysiological recordings in the OFC and DMS in freely moving rats. A large proportion of task-modulated single units were identified among all the units resolved in both brain areas (OFC: 1,157 of 1,329 units, *n* = 6 rats; DMS: 524 of 656 units, *n* = 6 rats). In both regions, trial-averaged single-unit activity spanned the trial, and single units that were modulated by a range of task features were identified (Fig. 3a,b). We observed striking similarity in neural encoding in the OFC and DMS, with single-unit responses dominated by the spatial features of the task (size of the reward offered on the left, size of the reward offered on the right, and side chosen) in both brain areas (Fig. 3c and Extended Data Fig. 4a). Interestingly, in agreement with our inactivation data, we observed that despite a similar proportion of neurons encoding both the objective value (size) and subjective value of rewards predicted by cues presented on either side of the animal, neurons in the OFC were more strongly modulated by the subjective value of a stimulus than by its objective value, an effect that was not observed in the DMS (Extended Data Fig. 4b).

To characterize the temporal dynamics of encoding between the OFC and DMS, we trained a linear support vector machine (SVM) to decode the choice the animal made on each trial (left or right) from neural activity data recorded in either the OFC or DMS (Fig. 3d). We were able to decode choice direction with high accuracy on held-out neural activity data from both brain regions. Importantly, across all animals, choice prediction peaked in the OFC before it peaked in the DMS (Fig. 3e and Extended Data Fig. 4c). We next examined how this temporal relationship related to choice accuracy. Cross-correlations of the predicted choice parameter (the perpendicular distance of the decoded decision value from the support vector, a proxy for decision confidence) computed on single trials revealed that the OFC led the DMS more on trials in which animals chose the larger available reward (‘correct’ trials) than on trials in which animals chose the smaller available reward (‘incorrect’ trials) (Fig. 3f,g; correct trials lag = −23.93 ± 22.86 ms (OFC leads), incorrect trials lag 44.82 ± 26.69 ms (DMS leads), *n* = 30 sessions from five rats). These data demonstrate that the encoding of choice-related information in the OFC precedes the encoding of choice-related information in the DMS, and that this relationship is correlated with choice accuracy.

**Extended Data Fig. 5:**
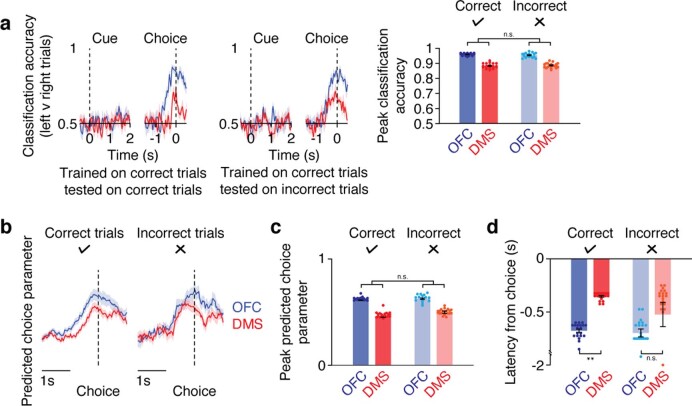
Electrophysiology supporting data 2. **a**. Chosen-side classification accuracy of 4-fold cross validated support vector machine trained on single unit activity recorded in either the OFC (blue) or DMS (red) on correct trials, and tested on either held-out correct trials (left) or incorrect trials (center). Note decoding performance is reduced compared to Fig. 3 due to the relatively small number of incorrect trials performed. Right, classification accuracy reaches equivalent levels on both correct and incorrect trials in both the OFC and DMS (n = 20 random samples, two-way repeated-measures ANOVA). **b**. Average predicted choice parameters computed on single correct (left) or incorrect (right) trials aligned to choice response (n = 30 sessions). **c**. Peak predicted choice parameters are equivalent on correct and incorrect trials (n = 20 random samples, two-way repeated-measures ANOVA). **d**. Latency to predicted choice parameter threshold relative to choice on correct or incorrect choice trials for models trained using data recorded simultaneously from OFC (blues) or DMS (reds). OFC activity does not precede DMS activity when animals make incorrect choices (n = 20 random samples, two-way repeated-measures ANOVA). * P < 0.05, ** P < 0.01, *** P < 0.001, Data are presented as mean ± SEM.

To examine how information transmission between the OFC and DMS might be disrupted on error trials, we first asked whether an SVM trained on trials where animals chose the larger available reward (correct trials) could predict choice behavior on trials when animals chose the smaller available reward (incorrect trials). Strikingly, a model trained on data recorded from either the OFC or DMS on correct trials predicted the side the animal would choose equally well on correct and incorrect trials (Extended Data Fig. 5a), suggesting that both brain areas encode the chosen side with equivalent accuracy regardless of the correctness of the choice. We next examined the SVM predicted choice parameters computed on held-out trials where the animal made either correct or incorrect choices. As before, on correct trials we observed that the predicted choice parameter increased in the OFC before the DMS. However, when animals made an erroneous choice, we observed that despite the predicted choice parameter reaching similar levels as seen on correct trials, the predicted choice parameter did not increase in the OFC before the DMS (Extended Data Fig. 5b–d). Thus, while the transmission of spatial choice information from the OFC to the DMS is necessary to initiate appropriate value-based choice behavior, without this information choice might be initiated by other brain regions reflecting internal biases relating to habitual behavior.

### Activity of the OFC projection to the DMS is necessary for economic decision-making

**Fig. 4:**
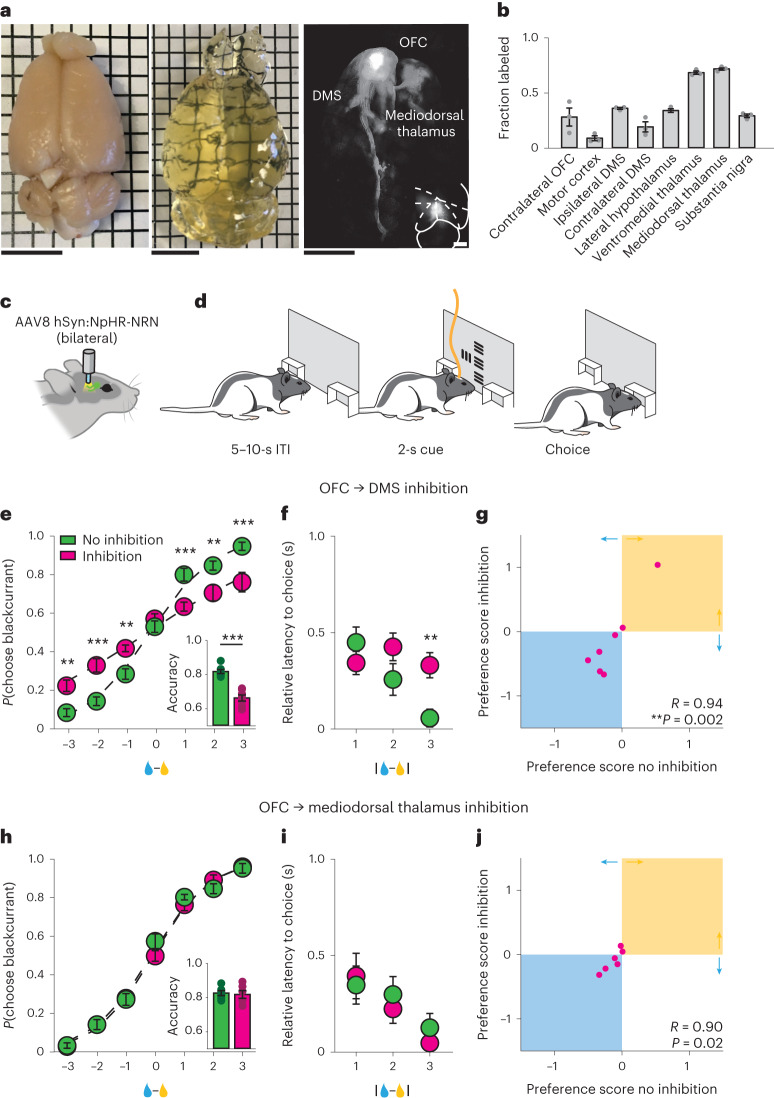
Activity of the projection from the OFC to the DMS is necessary for economic decision-making. **a**, Photograph of representative intact rat brain before (left) and after (middle) clearing. Right: brain-wide axonal projections of oScarlet-expressing cell bodies located in the OFC. Scale bar, 1 cm. Inset, coronal section of cell bodies located in the OFC. Scale bar, 1 mm. **b**, Quantification of brain-wide axonal projections of oScarlet-expressing cell bodies located in the OFC, *n* = 3 rats. **c**, Schematic of surgical preparation for inhibiting OFC axonal terminals during economic decision-making. **d**, Schematic of optical inhibition during the cue evaluation period of the economic decision-making task. **e**–**j**, Inhibiting the OFC projection to the DMS, but not the OFC projection to the mediodorsal thalamus, impairs economic decision-making. **e**,**h**, Probability of choosing the blackcurrant-predictive cue as a function of the difference in the volume of available rewards for uninhibited (green) and inhibited (magenta) trials. Rats were less likely to choose larger volume rewards when the OFC projection to the DMS was inhibited (**e**) but not when the OFC projection to the mediodorsal thalamus was inhibited (**h**) (OFC-DMS: *n* = 7 rats; OFC-mediodorsal thalamus: *n* = 6 rats, two-way repeated-measures ANOVA). Inset, fraction of trials in which the animal chose the larger available reward on uninhibited (green) and inhibited (magenta) trials (two-sided paired *t*-test). **f**,**i**, Latency to choice nosepoke response as a function of the absolute difference in the size of rewards available on uninhibited (green) and inhibited (magenta) trials. Rats were slower to respond when the difference in reward volume was high (easy trials), when the OFC projection to the DMS was inhibited (**f**). Inhibition of the OFC projection to the mediodorsal thalamus (**i**) did not alter response latency (OFC-DMS: *n* = 7 rats; OFC-MD: *n* = 6 rats, two-way repeated-measures ANOVA). **g**,**j**, Juice preferences computed on trials in which the OFC projection to the DMS (**g**) or mediodorsal thalamus (**j**) was inhibited were correlated with juice preferences computed on trials in which the OFC projection to the DMS or mediodorsal thalamus was not inhibited (Pearson correlation). **P < 0.01, ***P < 0.001. Data are presented as the mean ± s.e.m. Full statistical details are shown in Supplementary Table 1.

**Extended Data Fig. 6:**
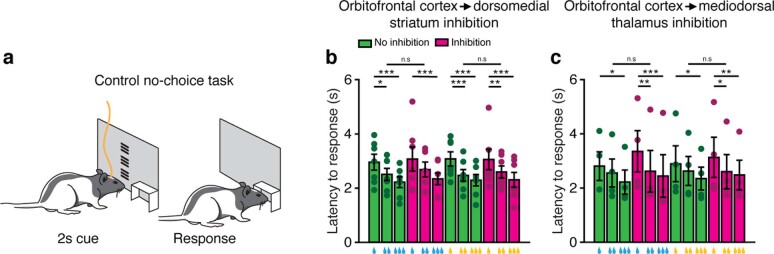
Optogenetic axon terminal inhibition control experiments, related to Fig. 4. **a**. Schematic of control task for probing whether effects of optically inhibiting OFC axon terminals specifically influence choice behavior. **b, c**. Latency to nosepoke response for cues predicting different size rewards in control no-choice task on trials in which the projection from OFC to DMS (**b**) or OFC to mediodorsal thalamus (**c**) was not inhibited (green) or was inhibited (magenta). Inhibition of OFC projections to DMS or mediodorsal thalamus does not alter latencies to respond in no-choice control task (n = 7 rats, two-way repeated-measures ANOVA). * P < 0.05, ** P < 0.01, *** P < 0.001, Data are presented as mean ± SEM.

The temporal relationship between choice-related information in the OFC and DMS suggests that choices represented in the OFC could be relayed to the DMS to guide appropriate choice behavior. To address this hypothesis, we first examined the axonal projections from the OFC and confirmed the presence of a robust projection to the DMS^26^ (Fig. 4a,b). We next specifically inhibited this direct projection by bilaterally injecting an AAV encoding a variant of the inhibitory halorhodopsin, which we optimized for axonal trafficking^27^, under the control of the human synapsin promoter (AA8 hSyn:eNpHR3.0-NRN-EYFP) into the OFC (Fig. 4c,d). We positioned optical fibers bilaterally in either the DMS or mediodorsal thalamus, another major target of the OFC projections (Fig. 4b). We found that optogenetic inhibition of OFC inputs into the DMS selectively impaired decision-making related to reward volume: psychometric curves were flatter and choice latencies were disrupted (Fig. 4e,f), while preference scores were unchanged, indicating that inhibition of the OFC projection to the DMS did not disrupt juice preferences (Fig. 4g). In contrast, optogenetic inhibition of the OFC inputs to the mediodorsal thalamus had no effect on economic decision-making (Fig. 4h–j). In addition, optogenetic inhibition of the OFC projection to the DMS or mediodorsal thalamus had no effect on response latencies in the control task in which the choice component of the economic decision-making task was selectively eliminated, confirming that this manipulation did not impair visual perception, action execution or the representation (or recollection) of cue value (Extended Data Fig. 6a–c). Taken together, the data shown in this study indicate that information relayed directly from the OFC to the DMS is important for guiding economic decision-making.

## Discussion

Animals must constantly evaluate stimuli in their environment to guide appropriate approach and avoidance behaviors^1–3^. To study how neural activity patterns across the brain may mediate these complex behaviors, we adapted an economic decision-making task for rats. Our experiments demonstrate that activity in the OFC and DMS, but surprisingly not in the prelimbic cortex or mediodorsal thalamus, is important for economic decision-making. Moreover, neural activity in both brain areas is dominated by spatial features of the economic decision-making task. Interestingly, we found that choice-related activity emerges in the OFC before the DMS, a relationship that correlates with choice accuracy. Finally, we found that activity of the direct connection from OFC to DMS is important for appropriate decision-making behavior. Taken together, these data suggest that spatial choice information is relayed from the OFC to the DMS to guide economic decision-making appropriate to the individual.

Several lines of previous evidence have supported a role for the OFC in economic decision-making^1,4–11^; however, inactivation and lesion studies have yielded contradictory results^12–16^. In this study, we leveraged the temporal resolution and enhanced the sensitivity of a designed inhibitory stabilized step function opsin^25^ to inhibit OFC selectively during the cue evaluation period, when rats are making decisions. This optogenetic strategy avoided prolonged tissue heating (which could modulate neural activity directly) and prevented OFC disruption during choice execution and reward consumption (which could have other influences on decision-making behavior^28,29^). In addition, we used a new training paradigm in which exposure to pairs of cues was limited to the testing context, so that animals would be unlikely to develop unnatural habitual responses to specific cue combinations (a phenomenon that could underlie the negative results observed in some previous studies^12–14^). This training paradigm resulted in precise psychometric curve functions that allowed us to detect subtle impairments in economic decision-making. Finally, we demonstrated that activity in the OFC was not necessary for performance of a control task in which the choice component was selectively removed. This experiment excluded the possibility that effects were driven by sensory, motor or motivational deficits induced by optical inhibition. Taken together, these data revealed that OFC inhibition—restricted to the cue evaluation period—specifically and potently impaired economic decision-making appropriate to individual preference.

The OFC has been proposed to function as a cognitive map of the world, that is, an internal model of the associative and predictive relationships present in the environment^30–34^. This hypothesis could unify several contrasting observations regarding the role of the OFC in distinct tasks, in which the OFC appears to be specifically required when individuals must use multiple categories of established knowledge to guide behavior in new scenarios^35–37^. Consistent with this hypothesis, we found that OFC activity is necessary when animals must choose between differently valued options, only previously experienced in isolation. Importantly, we observed that OFC inhibition does not appear to preclude the ability to access value information; for example, OFC-inhibited animals still respond more rapidly to cues that predict larger-magnitude rewards in a single-cue control condition. Notably, this is also a task the animals had never seen before.

These data therefore suggest that OFC activity (and associated cognitive maps) is specifically recruited when animals must resolve motivational conflict to guide new decision-making. It should be noted that the OFC is a large, heterogenous structure consisting of the medial, ventral, ventrolateral, lateral and dorsolateral orbital areas^33,38^. In this study, we specifically targeted the ventrolateral orbital area due to its reported role in supporting flexible behavior^39–42^. In the future, it will be important to determine how these results compare to inactivation of other orbitofrontal subregions and how future results relate to established differences in anatomical connectivity across mediolateral and anterior-posterior gradients^33,38^.

In contrast to previous observations of nonhuman primates making economic decisions^1,6^, which have consistently demonstrated that task variables are represented in the OFC in goods (that is, resource) space, our data suggest that the rodent OFC has a critical role in making decisions in action space^16,43^. Consistent with this idea, we observed that decision-related variables are represented in the rat OFC in a spatially mapped manner. Moreover, although optogenetic inhibition of the OFC did not influence behavior in animals presented with a single sensory cue eliciting a single action in the control task, optogenetic inhibition profoundly impaired behavior when animals were presented with the same single cue to guide decision-making between two different actions in the choice task (for example, three drops of blackcurrant juice reward versus no reward). Taken together, these data suggest that OFC activity in rodents is specifically recruited when animals must make choices between differently valued actions. Moving forward, it will be important to determine whether this reflects a fundamental difference in processing across species or is due to the different demands of the specific tasks used^44^ (for example, the freely moving task used in this study might necessitate a more detailed representation of the spatial environment than the head-restrained tasks that have typically been used in nonhuman primates).

In contrast to the role of the OFC itself, the role of OFC outputs to other brain regions in value-based decision-making has been less comprehensively characterized. Previous studies showed that OFC projections to the ventral tegmental area can mediate aspects of appropriate credit assignment^45^, projections to basolateral amygdala from lateral or medial OFC can mediate encoding and retrieval of values respectively^46,47^, and OFC projections to both the dorsal and ventral striatum are important for using outcomes to update the value of specific actions^42,48–53^. In this study, we expanded on this work and showed for the first time that the direct transmission of choice information from the OFC to the DMS, a region implicated in the generation of goal-directed actions^21,23,42,49,54–60^, is important for the evaluation of different reward options before any outcome is delivered. Moreover, by demonstrating that activity of the same projection is not required for performance of a control task in which we selectively removed motivational conflict, we confirm that this deficit in decision-making behavior is not due to a general failure to recall outcomes that specific cues predict^50^.

Surprisingly, while inhibition of the OFC disrupts choices based on both reward size (objective value) and reward type (subjective value), inhibition of either the DMS or the projection from the OFC to DMS only disrupts choices based on reward size (objective value). In addition, we found that neurons in the OFC are more strongly modulated by subjective value than objective value, an effect that is not observed in the DMS. These data suggest that an additional pathway out of the OFC may also contribute to decision-making about different types of reward. In the future, it will be important to identify how distinct OFC projections function in concert to support different components of decision-making. Taken together, these data provide new insight into how choices encoded in the OFC engage downstream neural circuits to generate appropriate behavioral responses.

Economic decision-making requires animals to compare the subjective value of sensory stimuli to guide appropriate behavior. To achieve this goal, sensory representations must be imbued with subjective value information, compared and used to engage neural circuits that generate appropriate behavioral responses. In this study we report that the projection from the OFC to the DMS ultimately connects sensory representations to appropriate behavioral output, to implement accurate economic decisions. Thus, the OFC projection to the DMS provides a critical anatomical substrate through which cortical representations exert dynamic control over ongoing behavior.

## Methods

Experimental procedures were approved by the Stanford University Institutional Animal Care and Use Committee and by the Administrative Panel on Laboratory Animal Care (protocol no. 32908), according to the National Institutes of Health (NIH) guidelines for the care and use of laboratory animals.

### Experimental animals and stereotactic surgery

Adult (10–12 weeks) male and female Long–Evans rats (Charles River Laboratories) were group-housed until surgery. Rats were randomly assigned to different experimental groups. Animals were anesthetized with isoflurane (1–5%, Henry Schein) and placed into a stereotactic frame (Kopf Instruments). Bone screws (Stoelting Co.) were inserted. For the optogenetic experiments, microinjection needles (WPI) were then inserted (coordinates from bregma: OFC +4 anteroposterior, ±2 mediolateral, −3 dorsoventral; prelimbic cortex +2.5 anteroposterior, ±0.5 mediolateral, −3.5 dorsoventral; DMS +1 anteroposterior, ±2.5 mediolateral, −4 dorsoventral; mediodorsal thalamus −2.8 anteroposterior, ±0.8 mediolateral, −5 dorsoventral; note that the dorsoventral coordinates reflect the distance from the brain surface) and each structure was injected with virus at a speed of 0.1 μl min. A 200-μm diameter optical fiber (Thorlabs) was placed 250 μm above the target sites and fixed in place using dental cement (RelyX, 3M). For the electrophysiological recordings, 64-channel silicon probes (Cambridge NeuroTech) were mounted on a microdrive and lowered to 500 μm above the site of interest. Craniotomies were sealed with Dura-Gel and microdrives were fixed in placed using dental cement. Molex connectors were attached to a wireless headstage (White Matter LLC), which was affixed to the skull with dental cement. Probes were lowered to the recording site 2 days before recordings. Buprenorphine SR (1 mg kg^−1^) was administered. As an exclusion criterion, we only included rats with viral expression confined to the site of interest and fiber placement above the target site. (This resulted in the exclusion of one animal.) All experiments were conducted according to approved protocols at Stanford University.

### Rat behavior

Water scheduled rats (1 h of water per day) were placed into a custom operant chamber equipped with three nosepoke portals mounted on a screen. The center portal was equipped with a lick spout for reward delivery. Entries into each nosepoke portal were detected by the breakage of an infrared beam and licks were detected using a capacitive touch sensor. (This was omitted for the electrophysiological recordings.) All events were controlled and recorded using custom MATLAB code using the MATLAB Support Package for Arduino and the Psychophysics Toolbox v.3 (ref. ^61^). For training, animals were placed into the operant chamber. One second after entering the center portal they were presented with a visual cue on one side of the center portal. The type of cue (vertical or horizontal drifting gratings) indicated the type of reward associated with the cue (zero calorie blackcurrant-flavored or lemon-flavored water); the number of squares that included the cue indicated the size of reward associated with the cue. Lemon-predictive and blackcurrant-predictive cues could be presented on either the left or right side of the animal, randomized for each trial. After 2 s, animals had to perform a nosepoke to the side the cue was presented to obtain the corresponding reward. Reward was delivered in the center portal. Reward collection was followed by a variable intertrial interval (ITI) of 5–10 s. If animals responded to the wrong side, no reward was delivered and the screen turned white for a 10-s time-out period. This taught animals to move to the side of the cue to indicate the response and to reinforce contingency. Trials in which animals took more than 12 s to indicate a response, and trials in which the animal took more than 5 s to collect the reward, were excluded.

When animals had achieved criterion performance (> 90% accuracy and response latency inversely proportional to reward magnitude on three consecutive sessions; each stimulus was comparably learned as shown in Extended Data Fig. 1a), they were placed into a full choice session. Animals were placed into the operant chamber; 1 s after entering the center portal, animals were presented with two visual cues side by side. Lemon-predictive or blackcurrant-predictive cues could be presented on either the left or right side of the animal, randomized for each trial. After 2 s, animals had to move to the side of the chosen cue to indicate their choice, and the chosen reward was delivered in the center portal. Reward collection was followed by a variable ITI of 5–10 s. Trials in which animals took more than 12 s to indicate choice, and trials in which animals took more than 5 s to collect the reward, were excluded. If animals performed at more than 75% accuracy (as animals made choices primarily to maximize the total volume of liquid consumed, accuracy was defined as the proportion of trials wherein animals selected the larger available reward), the following day animals were placed into another full choice session (for a maximum of three consecutive full choice sessions). For the choice sessions, a total of 15 cue combinations were used; each session was terminated after 600 trials or after 2.5 h, whichever came first. Otherwise, animals were placed back into training sessions until reachieving criterion performance. Summary data are presented as a composite of three consecutive full choice sessions per rat. Behavioral data were fitted by probit regression using the glmfit function in MATLAB. Preference scores were computed by calculating the difference in available reward (number of drops of blackcurrant − number of drops of lemon) for which the animal was equally likely to choose a blackcurrant-predictive or lemon-predictive cue. Long-term preference comparisons were between the preference score from the final three consecutive full choice sessions before a 4-month university shutdown, and the preference score from the first three consecutive full choice sessions after the 4-month shutdown. Comparisons of short-term preferences were performed on preference scores from each of three consecutive sessions. Latency to choice was calculated by finding the mean latency from the end of the mandatory 2-s cue presentation period, to the time at which the animal made its nosepoke response for each trial type. For each animal, we then subtracted the trial type with the fastest mean response time from all other trial types to obtain a relative latency to choice.

We carried out control behavior to account for the nonspecific effects of optical inhibition. Animals were placed into an operant chamber equipped with two nosepoke portals mounted on a screen; the left portal was equipped with a lick spout for reward delivery. One second after entering the left portal, animals were presented with a single visual cue in the center of the portal. (The same visual cues were also used for training and the full choice task.) After 2 s, animals had to perform a nosepoke in the second portal to indicate response. Reward was delivered in the left portal. Reward collection was followed by a variable ITI of 5–10 s. Trials in which animals took more than 12 s to indicate response, and trials in which the animal took more than 5 s to collect the reward, were excluded.

### Optogenetic inhibition

Rats were placed into the operant chamber and a top-branch with a 200-μm diameter fiber-optic patch cord (Doric) coupled to either a 473 nm (Omicron) and 635 nm (CNI), or a 594 nm (Cobalt), laser setup outside of the operant chamber connected to the implanted optical fibers. Immediately beforehand, power output from the patch cord was adjusted to 8 mW (473 nm), 5 mW (635 nm) or 10 mW (594 nm). Animals received randomly interleaved presentations of inhibited and uninhibited trials. On the SwiChR++ inhibition trials, 1 s of 473-nm light stimulation to initiate inhibition was delivered when the visual stimuli were presented; 1 s of 635 nm light stimulation to relieve inhibition was delivered when the animal exited the center portal to indicate its choice. On the halorhodopsin inhibition trials, 594-nm light stimulation was initiated when the visual stimuli were presented and terminated when the animal exited the center portal to indicate choice.

### Chronic electrophysiology

Animals were implanted with 64-channel silicon probes over the right DMS and right OFC. On the day of implantation, electrodes were lowered to 500 μm above the site of interest. Animals were allowed to recover for 2–3 weeks before behavioral training was resumed. Microdrives were lowered by 250 μm 2 days before each recording session. Electrophysiological data were acquired at 20 kHz using a wireless acquisition system (White Matter LLC). Recordings were made in freely moving rats, which may impact the degree of lateralization of the neural responses observed. Behavioral time stamps were acquired at 30 kHz using an Open Ephys acquisition system. Clocks were synchronized by sending a signal on every Open Ephys sample to the White Matter LLC acquisition system.

### Acute electrophysiology

Animals expressing SwiChR++ in the OFC were anesthetized with isoflurane and placed into a stereotactic frame. A craniotomy was placed over the OFC and a custom optrode (200-μm fiber cemented onto a silicone probe) was inserted into the region of the infected cells. Recordings were made using an Open Ephys acquisition system applying a bandpass filter from 300 to 6,000 Hz to the voltage signal. A 1-s pulse of blue light (473 nm, 8 mW) was delivered to initiate inhibition and a 1-s pulse of red light (635 nm, 5 mW) was delivered to alleviate inhibition 4 s later. Laser timing was controlled by a Master-8 pulse generator (AMPI).

### Electrophysiology data analysis

Spikes were sorted using Kilosort2 and were manually curated using Phy2 (ref. ^62^). Units with less than 1% inter-spike intervals shorter than 2 ms were considered single units for the analysis purposes. Spike counts were binned in 50-ms bins, stepped at 25-ms increments and converted into a *z*-scored firing rate across the whole session. *Z*-scored firing rates were aligned to task events (cue presentation, choice nosepoke and reward delivery) and the mean firing rate in the 500 ms before cue presentation was subtracted on a per trial basis. Task-modulated units were identified based on a Wilcoxon rank-sum test of the mean firing rate within the 500-ms baseline and ten 500-ms epochs spanning the trial starting at cue onset. A cell was deemed task-modulated if any of the task epochs differed significantly from baseline after false discovery rate correction, with a corrected significance threshold of *P* < 0.001. For each neural response, we performed a linear regression against each of a set of ten predefined variables (separately). For subjective value regression, preference scores were calculated for each session by finding the difference in the available reward at which the animal was equally likely to choose blackcurrant and lemonade. This score was then added to the volume of lemonade available on each trial to generate subjective value predictors. Units were deemed modulated by the variable if the regression slope differed significantly from zero (correct significance threshold of *P* < 0.001).

Decoding analysis was performed using a fourfold cross-validated linear SVM^63^. Classification accuracy was calculated as the fraction of correct predictions made on held-out data averaged across four cross-validation splits, repeated five times. For the single-trial analysis, predicted choice parameters were computed as the perpendicular distance of decision value from the support vector at each time point, repeated across four cross-validation splits. Cross-correlations of the predicted choice parameters were calculated in the 3 s surrounding the choice nosepoke and averaged across 20 decoding repeats per session. Single-trial predicted choice parameters were smoothed with a 50-ms Gaussian filter for analysis and a 250-ms Gaussian filter for visualization. For the latency analysis, an arbitrary threshold of 0.33 was set. For all decoding analysis, the numbers of units across brain areas were matched to the size of the smallest recorded population.

### Histological processing and analysis

Rats were euthanized by transcardiac perfusion with 150 ml PBS, followed by 100 ml 4% paraformaldehyde. Brains were extracted and 100-μm sections were cut on a vibratome. Slices were labeled with goat anti-GFP (1:1,000, Abcam) primary antibody and Alexa Fluor 488 donkey anti-goat (1:1,000, Invitrogen). For the axon tracing studies, a microinjection needle was inserted into the brain (coordinates from bregma: +4 anteroposterior, ±2 mediolateral, −3 dorsoventral) and 0.5 μl AAV8 hSyn:oScarlet was injected into the OFC at a speed of 0.1 μl min^−1^. At least 8 weeks later, brains were prepared for histology and axonal projections were quantified as described previously^64^. Briefly, 100-μm coronal slices were imaged on a confocal microscope (ZEISS, Zen software) using a ×20 objective and the resultant images were processed in ImageJ for quantification. Briefly, the injection site was first manually removed and background was subtracted. Threshold was set to ×4 the mean of the local background and pixels above this threshold were interpreted as positive signal from the OFC axons. Region of interest (ROI) boundaries were manually defined based on 4,6-diamidino-2-phenylindole staining and the Paxinos and Watson rat brain atlas^65^. Axon density was calculated as the percentage of total ROIs containing pixels above the threshold. Three sections per ROI were analyzed and those values were averaged to calculate a single value per ROI per rat. This approach cannot distinguish between axon terminal and fibers of passage.

### Whole-brain clearing

Adult Long–Evans rats were anesthetized with isoflurane and placed into a stereotactic frame. A microinjection needle was inserted into the brain (coordinates from bregma: +4 anteroposterior, ±2 mediolateral, −3 dorsoventral) and 0.5 μl AAV8 hSyn:oScarlet was injected into the OFC at a speed of 0.1 μl min^−1^. Eight weeks later, brains prepared for imaging using SHIELD^66^. Briefly, rats were euthanized by transcardial perfusion with 150 ml PBS, followed by 100 ml 4% paraformaldehyde, followed by 50 ml 12% epoxide SHIELD perfusion solution. Brains were extracted and incubated in SHIELD perfusion solution at 4 °C for 48 h. Brains were removed from SHIELD perfusion solution and transferred to SHIELD OFF solution and incubated at 4C for 48 h. Brains were then transferred to SHIELD ON solution and incubated at 37 °C for 24 h. After completion of the SHIELD reaction, brains were transferred to SDS clearing solution and cleared passively at 37 °C for 7 days, before being transferred to a SmartClear system for active clearing for 10–14 days. When brains were clear, they were washed in 0.1% PBS with Tween 20 at 37 °C for 3 days, before being equilibrated in exPROTOS at room temperature for 2 days and imaged using a COLM light sheet microscope^67,68^.

### Statistics and reproducibility

Data are presented as the mean ± s.e.m. unless otherwise indicated. Raw data were tested for normality of distribution; statistical analyses were performed using Student’s *t*-test, Wilcoxon signed-rank test or ANOVA with Bonferroni correction for multiple comparisons. Statistical analyses were performed in Prism (GraphPad Software) and MATLAB (MathWorks). No statistical method was used to predetermine sample size, but sample sizes were based on previous studies^69^. For practical reasons, data collection and analysis could not be performed blind to the conditions of the experiments (for example, because of obviously different positions of the fibers), but data were collected and analyzed in an automated manner to prevent experimenter bias.

### Reporting summary

Further information on research design is available in the Nature Portfolio Reporting Summary linked to this article.

## Online content

Any methods, additional references, Nature Portfolio reporting summaries, source data, extended data, supplementary information, acknowledgements, peer review information; details of author contributions and competing interests; and statements of data and code availability are available at 10.1038/s41593-023-01409-1.

### Supplementary information

Reporting Summary

Supplementary Table 1 Statistics summary. Full statistical details for each figure.

## Data Availability

All primary data for the figures and extended data figures are available from the corresponding author (K.D.) upon request.

## Code availability

The code used for data processing and analysis is available from the corresponding author (K.D.) upon request.
