# Supplementary material for: Orbitofrontal cortex control of striatum leads economic decision-making
Source: Nat Neurosci. 2023 Aug 17;26(9):1566–74. doi: 10.1038/s41593-023-01409-1 (PMC10471500; doi:10.1038/s41593-023-01409-1)
Supplement: Supplementary file 1 — Reporting Summary [file 41593_2023_1409_MOESM1_ESM.pdf]

nature portfolio

Corresponding author(s): Karl Deisseroth

Last updated by author(s): 06/19/2023

Reporting Summary

Nature Portfolio wishes to improve the reproducibility of the work that we publish. This form provides structure for consistency and transparency in reporting. For further information on Nature Portfolio policies, see our Editorial Policies and the Editorial Policy Checklist.

Statistics

For all statistical analyses, confirm that the following items are present in the figure legend, table legend, main text, or Methods section.

n/a	Confirmed	
☐	☒	The exact sample size (*n*) for each experimental group/condition, given as a discrete number and unit of measurement
☐	☒	A statement on whether measurements were taken from distinct samples or whether the same sample was measured repeatedly
☐	☒	The statistical test(s) used AND whether they are one- or two-sided<br>*Only common tests should be described solely by name; describe more complex techniques in the Methods section.*
☐	☒	A description of all covariates tested
☐	☒	A description of any assumptions or corrections, such as tests of normality and adjustment for multiple comparisons
☐	☒	A full description of the statistical parameters including central tendency (e.g. means) or other basic estimates (e.g. regression coefficient) AND variation (e.g. standard deviation) or associated estimates of uncertainty (e.g. confidence intervals)
☐	☒	For null hypothesis testing, the test statistic (e.g. *F*, *t*, *r*) with confidence intervals, effect sizes, degrees of freedom and *P* value noted<br>*Give P values as exact values whenever suitable.*
☒	☐	For Bayesian analysis, information on the choice of priors and Markov chain Monte Carlo settings
☒	☐	For hierarchical and complex designs, identification of the appropriate level for tests and full reporting of outcomes
☒	☐	Estimates of effect sizes (e.g. Cohen's *d*, Pearson's *r*), indicating how they were calculated

Our web collection on statistics for biologists contains articles on many of the points above.

Software and code

Policy information about availability of computer code

Data collection	Open Ephys and White Matter (in vivo extracellular electrophysiology acquisition), Matlab 2019b and Psychtoolbox3 (behavioral control and acquisition), Zen (confocal image acquisition).
Data analysis	Matlab 2019b, GraphPad Prism 9, Kilosort2, Phy2, ImageJ.

For manuscripts utilizing custom algorithms or software that are central to the research but not yet described in published literature, software must be made available to editors and reviewers. We strongly encourage code deposition in a community repository (e.g. GitHub). See the Nature Portfolio guidelines for submitting code & software for further information.

Data

Policy information about availability of data

All manuscripts must include a data availability statement. This statement should provide the following information, where applicable:

- Accession codes, unique identifiers, or web links for publicly available datasets
- A description of any restrictions on data availability
- For clinical datasets or third party data, please ensure that the statement adheres to our policy

The data that support this paper are available upon request to the corresponding author.

Research involving human participants, their data, or biological material

Policy information about studies with human participants or human data. See also policy information about sex, gender (identity/presentation), and sexual orientation and race, ethnicity and racism.

Reporting on sex and gender	N/A
Reporting on race, ethnicity, or other socially relevant groupings	N/A
Population characteristics	N/A
Recruitment	N/A
Ethics oversight	N/A

Note that full information on the approval of the study protocol must also be provided in the manuscript.

Field-specific reporting

Please select the one below that is the best fit for your research. If you are not sure, read the appropriate sections before making your selection.

☒ Life sciences ☐ Behavioural & social sciences ☐ Ecological, evolutionary & environmental sciences

For a reference copy of the document with all sections, see nature.com/documents/nr-reporting-summary-flat.pdf

Life sciences study design

All studies must disclose on these points even when the disclosure is negative.

Sample size	No statistical methods were used to determine sample size a priori. Sample sizes were based on prior studies (Zalocusky et al., 2016).
Data exclusions	Animals with unintended experimental error (poor ChR2, fiber, or electrode placement) were excluded.
Replication	Data was collected in 3 or more experiments and all results were pooled. All attempts at replication were successful
Randomization	Rats were randomly assigned to each experimental group.
Blinding	Blinding of group was not possible due to obvious differences in fiber position. However data were collected and analyzed in an automated manner to prevent experimenter bias.

Reporting for specific materials, systems and methods

We require information from authors about some types of materials, experimental systems and methods used in many studies. Here, indicate whether each material, system or method listed is relevant to your study. If you are not sure if a list item applies to your research, read the appropriate section before selecting a response.

Materials & experimental systems

n/a	Involved in the study	
☐	☒	Antibodies
☒	☐	Eukaryotic cell lines
☒	☐	Palaeontology and archaeology
☐	☒	Animals and other organisms
☒	☐	Clinical data
☒	☐	Dual use research of concern
☒	☐	Plants

Methods

n/a	Involved in the study	
☒	☐	ChIP-seq
☒	☐	Flow cytometry
☒	☐	MRI-based neuroimaging

Antibodies

Antibodies used	Antibodies used Goat anti-GFP (Abcam ab6673, 1:1000). Alexa Fluor 488 anti-goat (Life Technologies A11055, 1:1000).
Validation	Validation and references on manufacturer's website:

Validation	ab6673: anti-GFP assayed by ELISA for direct binding of antigen recognizes wild type, recombinant and enhanced forms of GFP. IHC. Species independent. 483 references.<br>A11055: IHC tested 1-10ug/ml. 2866 references.

Animals and other research organisms

Policy information about studies involving animals; ARRIVE guidelines recommended for reporting animal research, and Sex and Gender in Research

Laboratory animals	Charles River and in-house bred Long Evans rats, 10-12 weeks.
Wild animals	No wild animals were used in this study.
Reporting on sex	Data were collected from male and female rats. No differences were observed between male and female animals so results were pooled.
Field-collected samples	No field samples were collected in this study.
Ethics oversight	Experimental procedures were approved by the Stanford University Institutional Animal Care and Use Committee (IACUC) and by the Administrative Panel on Laboratory Animal Care (APLAC), following the National Institutes of Health guidelines for the care and use of laboratory animals.

Note that full information on the approval of the study protocol must also be provided in the manuscript.
